# A Robust 3D Registration Method via Simultaneous Inlier Identification and Model Estimation

**DOI:** 10.3390/jimaging12060247

**Published:** 2026-06-01

**Authors:** Xianyun Qian, Fei Wen, Peilin Liu

**Affiliations:** School of Integrated Circuits, Shanghai Jiao Tong University, Shanghai 200240, China; wenfei@sjtu.edu.cn (F.W.); liupeilin@sjtu.edu.cn (P.L.)

**Keywords:** robust fitting, 3D registration, geometric transformation

## Abstract

Robust 3D registration is a fundamental problem in computer vision and robotics, where the goal is to estimate the geometric transformation between two sets of measurements in the presence of noise and outlier contamination. Existing robust registration methods are mainly built on either maximum consensus (MC) estimators, which first identify inliers and then estimate the transformation, or M-estimators, which directly optimize a robust objective. However, MC-based methods typically ignore residual magnitudes during inlier selection, while many M-estimators do not explicitly couple inlier/outlier identification with model estimation. Thus, a unified and efficient framework that jointly performs inlier identification and accurate transformation estimation remains desirable for challenging 3D registration. In this work, we introduce a unified truncated-loss based formulation for simultaneous inlier identification and model estimation (SIME) and study it in the context of 3D registration. We show that, compared with MC-based robust fitting, SIME can achieve a lower fitting residual because it incorporates residual magnitudes into the inlier selection process. To solve the resulting nonconvex problem, we develop an alternating minimization (AM) algorithm, and further propose an AM method embedded with semidefinite relaxation (AM-R) to alleviate the difficulty caused by the binary inlier variables. We instantiate the proposed framework for 3D rotation search and rigid point-set registration using quaternion-based formulations. Experimental results on both simulated and real-world registration tasks demonstrate that the proposed methods compare favorably with strong baseline solvers, especially in high noise and extreme outliers. In the synthetic experiments, the proposed methods are evaluated under outlier ratios up to 95% and consistently achieve competitive or better accuracy, with clear advantages in high-noise cases. On 3DMatch, SIME (AM) achieves a mean registration success rate of 91.0%. These results show the potential of SIME for reliable 3D registration in practical robotics, computer vision, and geometric perception applications.

## 1. Introduction

Robust 3D registration is a central problem in computer vision, robotics, and geometric perception [1,2,3]. Given a set of tentative correspondences between two 3D data sets, the goal is to estimate the underlying geometric transformation, such as a 3D rotation or a full rigid motion, despite the presence of measurement noise and outlier correspondences. Robust 3D registration is a key component in a wide range of applications, including point cloud alignment, object pose estimation, robot localization, and multi-view reconstruction.

A major challenge in 3D registration lies in the large number of outliers that often arise in practical correspondence generation. For example, feature matching between point clouds or scans can easily produce incorrect correspondences due to repetitive structures, partial overlap, occlusion, or sensor noise. As a result, standard least-squares estimation is highly sensitive to mismatches, and robust estimators are required.

Existing robust registration methods can be broadly divided into several categories. Maximum consensus (MC) methods aim to find a transformation that is consistent with as many correspondences as possible under a prescribed inlier threshold. Classic RANSAC [4] and its variants [5,6,7,8,9,10,11] are representative randomized MC methods and remain widely used due to their simplicity and broad applicability. Recent consensus and graph-based methods further improve hypothesis generation and outlier rejection by exploiting geometric compatibility among correspondences, such as maximal-clique based registration, deterministic sampling under extreme outlier ratios, and heuristic consensus sampling [12,13]. Global MC methods based on branch-and-bound, tree search, or enumeration [14,15,16,17,18,19] can provide optimality guarantees, while deterministic approximate MC methods, such as convex relaxation, reweighted $\ell_{1}$, $\ell_{0}$, and exact penalty methods [20,21,22,23,24], provide more efficient alternatives.

Another important line of work is robust optimization and M-estimation, where the transformation is estimated by directly minimizing a robust loss function [25,26,27,28,29]. Among them, truncated-loss based methods are particularly relevant to robust registration, since they combine robustness to large outliers with the ability to suppress incorrect correspondences. Recent examples include semidefinite relaxation for robust rotation estimation [30], graduated nonconvexity for scalable registration [28], certifiable schemes for rigid point cloud registration [31], and scalable truncated-residual formulations for large-scale correspondence sets [32]. In parallel, learning-based methods have been developed to learn more reliable descriptors, correspondences, or outlier scores. Representative recent works include variational Bayesian outlier rejection [33], methods balancing accuracy, efficiency, and generalization [1], transformer-based large-scale registration [34], and dynamic-cue and focus-attention based matching [2,35].

Despite this progress, several limitations remain. MC and graph-compatibility methods are effective for rejecting gross outliers, but they mainly rely on hard inlier counting, sampling, or compatibility thresholds. As a result, two transformations with the same consensus size but very different fitting residuals may be treated similarly. Globally optimal or certifiable methods can provide stronger guarantees, but their computational cost or problem-specific relaxation often limits scalability. Robust M-estimators and truncated-loss methods improve robustness, but many of them focus primarily on parameter estimation or require a tailored solver for a specific registration setting. Learning-based methods can produce high-quality correspondences, but their performance may depend on the training distribution, descriptor quality, and post-processing robust estimator. These observations motivate an optimization-level formulation that directly couples inlier identification with model fitting, while remaining applicable to both rotation-only and full Euclidean registration.

In this paper, we investigate a truncated-loss formulation for simultaneous inlier identification and model estimation (SIME) for robust 3D registration. Unlike MC-based robust fitting, which first searches for a large consensus set and then estimates the model, SIME jointly determines which correspondences should be treated as inliers and what transformation best explains them. The key difference is that SIME incorporates residual magnitudes into the inlier selection process. We show that, under the same truncation threshold, SIME can achieve a lower fitting residual than MC-based robust fitting. This property is especially useful for 3D registration, where the quality of the transformation estimate and the correctness of the inlier set are strongly coupled.

To solve the resulting nonconvex problem, we develop two alternating minimization algorithms. The first one is a direct AM algorithm that alternates between binary inlier-variable update and transformation update. The second one, referred to as AM-R, embeds a semidefinite relaxation (SDR) of the binary variables into the AM framework to alleviate the difficulty caused by the combinatorial inlier selection. To improve scalability, we further exploit the sparsity of the resulting SDP subproblem and use a low-rank Burer–Monteiro factorization, which substantially reduces the number of optimization variables. We instantiate the proposed framework for both 3D rotation search and 6-DoF rigid registration using quaternion-based formulations.

We validate the proposed methods on both synthetic and real-world registration tasks. The synthetic experiments include 3-DoF rotation registration and 6-DoF Euclidean registration under different noise levels and outlier ratios. The real-world experiment is conducted on the 3DMatch scan matching benchmark, where the proposed method is compared with representative robust registration solvers.

The experimental results show that the proposed methods compare favorably with strong baselines, especially in challenging cases with high noise levels and many outliers. In the synthetic rotation experiments, the proposed methods are evaluated with outlier ratios up to 95%, and their advantage is particularly clear in high-noise settings. On 3DMatch, SIME (AM) achieves the best mean registration success rate among the compared methods.

Our main contribution is a unified SIME framework, rather than the separate use of truncated losses, quaternion parametrization, or SDR. In contrast to MC-based methods, which select inliers only based on a residual threshold, our SIME formulation couples inlier identification with model fitting. This leads to a different criterion and we prove that SIME can achieve a lower fitting residual than MC. Compared with existing truncated-loss based methods such as QUASAR [30], our framework is more general: while QUASAR corresponds to a special squared-loss instance of SIME, our formulation accommodates generalized truncated losses. Moreover, our proposed AM-R algorithm relaxes the binary inlier variables directly and exploits the sparse low-rank structure of the resulting SDP, which leads to a tighter and more scalable relaxation than the SDR used in QUASAR.

Notations: I(·)∈{0,1} is the indicator function. ⊗ and ∘ stand for the Kronecker product and quaternion product, respectively. $1_{N}$ is an *N* dimension vector with all elements being unit, and 0 is a zero vector or matrix with a proper size. $∥\cdot ∥$ and ${∥\cdot ∥}_{p}$ denote the Euclidean and $\ell_{p}$ norm, respectively. ${\left(\cdot \right)}^{T}$ denotes transpose. $S^{N}$ is the set of N×N real-valued symmetric matrices. For $X\in S^{N}$, X⪰0 means that X is semidefinite. Both X(i,j) and $X_{i,j}$ denote the (i,j)-th element of the matrix X. vec(·) is the vectorization operation of a matrix.

## 2. Methodology

### 2.1. Robust Fitting Using Truncated Loss

Traditionally, maximum consensus (MC) methods are widely used for 3D registration to first identify inliers and then fit a model on the inliers. Here we consider simultaneous inlier identification and model estimation (SIME) using truncated loss. We first compare these two criteria and then instantiate SIME for 3D registration.

#### 2.1.1. Simultaneous Inlier Identification and Model Estimation Using Truncated Loss

Truncated loss based M-estimation can be traced back to the studies [36,37,38] where truncated LS loss is used to construct robust estimators with influence functions going to zero. Recently, truncated LS loss has been used to develop robust algorithms for rotation searching [30] and point cloud registration [31], which can tolerate extreme amounts of outliers.

Given a set of *N* measurements, let Φ denote a generalized loss function to be truncated and β denote the truncating level. Using such a truncated loss to estimate a mode parameterized by $\theta \in R^{d}$, the SIME formulation considered in this work is

(1) $$\begin{array}{cc} \; & \underset{\theta \in R^{d},s\in {\left\{0,1\right\}}^{N}}{\min\;}\sum_{i=1}^{N}\left(1\;-\;s_{i}\right)\Phi \left(r_{i}\left(\theta \right)\right)+\beta s_{i}\\ \; & \;\;\;\;\;\;\;\;\text{s.t.}\;\;\;\;\;h(\theta )=0, \end{array}$$

where $r_{i}\left(\theta \right)$ gives the non-negative residual of the *i*-th measurement with respect to θ. h(θ)=0 is a constraint on the model parameter. For example, as will be detailed in Section 2.3, a constraint ∥θ∥=1 is present in rotation registration and Euclidean registration problems, in which case h(θ)=∥θ∥−1. The objective in (1) is a truncated cost as $\min_{s_{i}\in \left\{0,1\right\}}\left(1\;-\;s_{i}\right)\Phi \left(\cdot \right)+\beta s_{i}$ is equivalent to $\min\left(\Phi (\cdot ),\beta \right)$ [30]. When Φ is the LS loss, problem (1) without the constraint h(θ)=0 becomes the minimum truncated LS formulation [37].

Note that we consider a generalized truncated loss rather than the truncated LS loss. While the LS loss is optimal for Gaussian noise, a generalized Φ also adapts to $\ell_{p}$ loss with p<2, which is desirable when the inlier noise is not Gaussian but super-Gaussian.

A feature of SIME is it can identify inliers/outliers based on the solution s, which is similar to the MC criterion. Given a set of *N* measurements, MC aims to find a feasible model parameterized by θ, that is consistent with as many of the measurements as possible up to an inlier residual threshold τ>0, i.e., has the largest consensus set *I* as [39]

(2) $$\begin{array}{cc} \; & \;\;\;\;\;\;\;\;\underset{\theta \in R^{d},I\subseteq \Omega }{\max\;}\left|I\right|\\ \text{s.t.}\; & r_{i}\left(\theta \right)\leq \tau ,\;\;\forall i\in I,\;\;h\left(\theta \right)=0, \end{array}$$

where Ω={1,2,…,N} is the index set of the measurements. Let $I^{*}$ be a solution of (2), then $I^{*}$ stands for the index set of the true inliers, while the complementary set of $I^{*}$, denoted by $\Omega \setminus I^{*}$, stands for the index set of the true outliers. Using some auxiliary binary variables $s={\left[s_{1},\ldots ,s_{N}\right]}^{T}\in {\left\{0,1\right\}}^{N}$, (2) can be reformulated into

(3) $$\begin{array}{cc} \; & \;\;\;\;\;\;\;\;\underset{\theta \in R^{d},s\in {\left\{0,1\right\}}^{N}}{\min\;}\sum_{i=1}^{N}s_{i}\\ \text{s.t.} & \;r_{i}\left(\theta \right)\leq \tau +s_{i}L,\;\;\forall i\in \Omega ,\;\;h\left(\theta \right)=0, \end{array}$$

where *L* is a sufficiently large positive constant.

The similarity and difference between SIME and MC can be observed from (1) and (3). Similar to SIME, for a solution of MC, the *i*-th measurement is identified as an outlier if $s_{i}=1$, or an inlier if $s_{i}=0$. While MC does not take the inlier residual into account in inlier identification, SIME takes that into consideration via simultaneous inlier identification and model fitting in a single step. Besides, unlike the θ solution of MC being only a feasible solution satisfying the residual constraint, the θ solution of SIME is the desired model fitting result on the identified inliers.

SIME uses β to balance the number of outliers against the fitting residual, by which the residual constraint in MC is removed. Obviously, a larger β would result in a larger number of $s_{i}$ values being zero, i.e., more identified inliers (which are not necessary to be true inliers). That is, a too large β would yield a larger consensus size than $I^{*}$ and include some true outliers, while a too small β would yield a smaller consensus size than $I^{*}$ and exclude some true inliers. As SIME estimates the model only using the measurements with residuals less than β, β can be easily selected as a threshold that tightly upper bounds the fitting residual of the true inliers. For example, if there exists a proper bound τ such that $r_{i}\left(\theta \right)\leq \tau$ for $\forall i\in I^{*}$ with a high probability for the true model, it is reasonable to choose β=Φ(τ).

The following result shows SIME can be viewed as an approximate maximum likelihood (ML) estimator under certain distribution assumptions of inliers and outliers.

**Proposition** **1.** *Denote $r_{i}=r_{i}\left(\theta \right)$ for succinctness. SIME admits an approximate maximum-likelihood interpretation under the following conditions: (i) For any true inlier, i.e., $\forall i\in I^{*}$, the residual density has the form*

$$p_{\mathrm{in}}\left(r_{i}|\theta \right)=c_{i}\exp\;\left(-\Phi (r_{i},\sigma_{i})\right),\;r_{i}\geq 0,$$

*where $c_{i}>0$ is a normalizing constant, $\sigma_{i}>0$ is a scale parameter, and $\Phi (\cdot ,\sigma_{i})$ is nondecreasing on [0,+∞). The model parameter is constrained by h(θ)=0. (ii) For any true outlier, i.e., $\forall i\in \Omega \setminus I^{*}$, the residual $r_{i}$ is approximately uniformly distributed on [τ,u], where u>τ is an upper bound on the outlier perturbation (the possible maximal perturbation of outliers).*

Proof of Proposition 1 is given in Appendix A.

**Remark** **1.** *This proposition suggests that SIME admits an approximate maximum-likelihood interpretation when the inlier residuals are relatively small and their density can be written in the form $p_{\mathrm{in}}\left(r_{i}\right)\propto \exp\left(-\Phi \left(r_{i},\sigma_{i}\right)\right)$, while the outlier residuals are approximately uniformly distributed over a bounded interval. This model covers a broad class of residual distributions. For example, when the inlier residual model is induced by Gaussian noise, *Φ *reduces to a quadratic form $\Phi \left(r_{i},\sigma_{i}\right)=\frac{r_{i}^{2}}{2\sigma_{i}^{2}}+\frac{1}{2}\log\left(2\pi \sigma_{i}^{2}\right)$, corresponding to the least-squares loss up to additive constants. It also accommodates super-Gaussian noise, for which $\Phi \left(r_{i}\right)\propto r_{i}^{p}$ with p<2, indicating that an $\ell_{p}$ loss is more appropriate in the case the inlier noise is super-Gaussian.*

#### 2.1.2. Comparison Between MC and SIME

This subsection compares MC with SIME to show that they can produce the same inlier set in special conditions, but not in general conditions. Particularly, SIME has lower fitting residual than MC fitting.

Before proceeding to the results, we present some definitions will be used in the analysis. Denote

$$\theta_{I}:=\arg\underset{\theta \in R^{d},h\left(\theta \right)=0}{\min\;}\sum_{i\in I}\Phi (r_{i}\left(\theta \right)),$$

$$R\left(I\right):=\sum_{i\in I}\Phi (r_{i}\left(\theta_{I}\right))=\underset{\theta \in R^{d},h\left(\theta \right)=0}{\min\;}\sum_{i\in I}\Phi (r_{i}\left(\theta \right)),$$

and the objective of SIME as

$$f\left(\theta ,s\right):=\sum_{i=1}^{N}\left(1-s_{i}\right)\Phi \left(r_{i}\left(\theta \right)\right)+\Phi \left(\tau \right)s_{i}.$$

As the objective of SIME is separable, for any fixed θ, the optimal choice of each binary variable is given by

$$s_{i}=\arg\min_{s\in \{0,1\}}\left(1-s\right)\Phi \left(r_{i}\left(\theta \right)\right)+\Phi \left(\tau \right)s.$$

Hence, for any global solution $\left(\theta^{⋆},s^{⋆}\right)$ of SIME with inlier set

$$I^{⋆}:=\left\{i:s_{i}^{⋆}=0\right\},$$

it necessarily holds that

$$\Phi \left(r_{i}\left(\theta^{⋆}\right)\right)\leq \Phi \left(\tau \right),\;\forall i\in I^{⋆},$$

and

$$\Phi \left(r_{i}\left(\theta^{⋆}\right)\right)\geq \Phi \left(\tau \right),\;\forall i\in \Omega \setminus I^{⋆}.$$

Accordingly, we define the collection of self-consistent inlier sets associated with SIME as

$$\begin{array}{c} Ϝ:=\{I\subseteq \Omega :\;\Phi \left(r_{i}\left(\theta_{I}\right)\right)\leq \Phi \left(\tau \right),\;\forall i\in I;\\ \;\Phi \left(r_{i}\left(\theta_{I}\right)\right)\geq \Phi \left(\tau \right),\;\forall i\in \Omega \setminus I\}. \end{array}$$

The set Ϝ contains the inlier sets that are self-consistent with the model estimated from themselves. That is, if we estimate the model using an inlier set *I*, then all correspondences in *I* should still have residuals below the truncation threshold, while the correspondences outside *I* should remain above the threshold. Thus, Ϝ characterizes the candidate inlier sets that can be stable under the SIME criterion. For any I⊆Ω, define

$$s_{I}:={\left[I\left(1\notin I\right),I\left(2\notin I\right),\ldots ,I\left(N\notin I\right)\right]}^{T}.$$

Then, $\left(\theta_{I},s_{I}\right)$ is a candidate support set that can correspond to a global SIME optimum only when I∈Ϝ. That is I∈Ϝ is a necessary self-consistency condition for *I* to be the inlier set of a global SIME solution.

The following result gives a sufficient condition for the two criteria to produce the same consensus set.

**Proposition** **2.** *Suppose that β=Φ(τ), and that *Φ* is increasing on [0,+∞) with Φ(0)=0. Let $I^{*}$ denote a solution of MC, and let $I^{+}$ denote the inlier set corresponding to a global solution of SIME. Then $I^{+}$ is a consensus set and $|I^{+}\left|\leq \right|I^{*}|$. In particular, if $\Phi \left(r_{i}\left(\theta_{I^{*}}\right)\right)\leq \Phi \left(\tau \right)$ for all $i\in I^{*}$, and for any other consensus set $I^{•}\in Ϝ$ there holds*

(4) $$|I^{*}\left|-\right|I^{•}|>\frac{R\left(I^{*}\right)-R\left(I^{•}\right)}{\Phi (\tau )},$$

*then $I^{+}=I^{*}$.*

**Remark** **2.** *Another special condition for $I^{+}=I^{*}$ in the case of truncated LS loss is given in Lemma 23 in [31]. Since $I^{*}$ is a largest consensus set, we have $|I^{*}\left|\geq \right|I^{•}|$ for any other consensus set $I^{•}$. Hence, with the choice β=Φ(τ), SIME yields the same inlier set as MC if the fitting residual over $I^{*}$ is sufficiently small (or relatively small compared with that over any other consensus set $I^{•}$), and if $\Phi \left(r_{i}\left(\theta_{I^{*}}\right)\right)\leq \Phi \left(\tau \right)$ for all $i\in I^{*}$. In general, however, SIME and MC do not necessarily produce the same consensus set. Moreover, a sufficient, though not necessary, condition for $I^{+}\neq I^{*}$ is that $\Phi \left(r_{i}\left(\theta_{I^{*}}\right)\right)>\Phi \left(\tau \right)$ for some $i\in I^{*}$, since in this case $I^{*}\notin Ϝ$, and hence $I^{*}$ cannot be the inlier set of a global SIME solution.*

Proof of Proposition 2 is given in Appendix B.

Since SIME takes the fitting residual into account in inlier selection, whereas MC does not, a natural question arises:

*How do SIME and MC differ in fitting performance?*

We answer this question by comparing the lowest achievable fitting residual under the two criteria.

**Theorem** **1** (SIME can achieve lower fitting residual than MC)**.**
*Let $I^{+}$ denote the inlier set corresponding to a global solution of SIME, and let $I^{*}$ denote a solution of MC. Suppose that *Φ* is increasing on [0,+∞) with Φ(0)=0. Then, with β=Φ(τ), the lowest achievable residual of SIME is no larger than that of MC:*

(5) $$\min_{\theta \in R^{d},h\left(\theta \right)=0}\sum_{i\in I^{+}}\Phi \left(r_{i}\left(\theta \right)\right)\leq \min_{\theta \in R^{d},h\left(\theta \right)=0}\sum_{i\in I^{*}}\Phi \left(r_{i}\left(\theta \right)\right).$$

*Furthermore, if $I^{*}\notin Ϝ$, then the inequality in (5) holds strictly.*

Proof of Theorem 1 is given in Appendix C.

**Remark** **3.** *This theorem highlights a fundamental difference between SIME and MC in terms of the lowest achievable fitting residual. In particular, SIME can attain a lower fitting residual than MC because it takes the fitting residual into account during inlier selection. However, this does not imply that SIME is universally preferable to MC. In some applications, the primary goal is to identify and remove outliers, while model estimation is of secondary importance (e.g., segmentation tasks). From this perspective, SIME should be regarded as a valid alternative to MC, which is maybe preferred for the applications of robust model fitting.*

The practical implication of the above result is that SIME is more directly aligned with the registration accuracy than pure consensus maximization. Specifically, for 3D registration, the goal is not only to retain many correspondences, but also to estimate a transformation with small geometric residuals. MC treats all correspondences below the residual threshold equally. In contrast, SIME favors inlier sets with smaller residuals after model fitting. Therefore, SIME can produce a cleaner effective inlier set and more accurate transformation estimation, especially in high-noise and/or high-outlier scenarios.

### 2.2. Efficient Alternating Minimization Algorithms for Solving the SIME Formulation

For the nonconvex SIME formulation (1), a natural optimization scheme is to update the variables θ and s in an alternating manner. However, since the problem is highly nonconvex involving binary variables, the direct AM algorithm can easily get trapped in local minima. To address this problem, we additionally propose an algorithm using SDR on the binary variable s.

#### 2.2.1. Direct Alternating Minimization Algorithm

The AM algorithm solves (1) via alternatingly updating the model parameter θ and the binary slack variable s. First, fixing θ, the s-subproblem can be solved explicitly as

(6) $$s_{i}=\left\{\;\begin{array}{cc} \;1, & \Phi (r_{i}\left(\theta \right))>\beta \\ \;0, & \Phi (r_{i}\left(\theta \right))\leq \beta \end{array}\right.\;,\;\;\;\;1\leq i\leq N.$$

Then, fixing s, the θ-subproblem is given by

(7) $$\underset{\theta \in R^{d}}{\min\;}\sum_{i=1}^{N}\left(1\;-\;s_{i}\right)\Phi \left(r_{i}\left(\theta \right)\right),\;\;\;\text{s.t.}\;\;\;h\left(\theta \right)=0,$$

which depends on the residual models of specific applications. We consider two main cases, a linear or nonlinear residual model.

Consider a linear residual model, which typically has a form of

(8) $$r_{i}\left(\theta \right)=\left|a_{i}^{T}\theta -b_{i}\right|.$$

In robust linear regression and many computer vision applications, the residual $r_{i}\left(\theta \right)$ can be conveniently expressed in this form. With a linear residual model, if Φ is chosen as the $\ell_{p}$ loss, the model fitting term in SIME becomes

(9) $$\Phi \left(r_{i}\left(\theta \right)\right)=|a_{i}^{T}\theta -b_{i}{|}^{p}.$$

For Gaussian inlier noise, p=2 is the optimal choice, while for super-Gaussian inlier noise, the optimal value of *p* should be p<2. Generally, a smaller *p* should be used when the inlier noise distribution has a thicker tail. Since the outliers are modeled as a wide uniform distribution (Proposition 1), it is reasonable to assume that the inlier noise is not too impulsive and hence we can restrict p≥1, in which case Φ is convex. Therefore, in this paper, we mainly consider truncated $\ell_{p}$ loss with p≥1.

For the $\ell_{p}$ model fitting loss (9) with p=2, the θ-subproblem (7) with or without the constraint ∥θ∥=1 can be solved explicitly. However, when 1≤p<2, the θ-subproblem (7) cannot be solved in closed-form. In this case, we solve it by the iteratively reweighted LS algorithm as

(10) $$\theta^{k+1}=\arg\min_{\theta \in R^{d}}\sum_{i=1}^{N}\left(1-s_{i}\right)w_{i}^{k+1}{\left(a_{i}^{T}\theta -b_{i}\right)}^{2},$$

subject to h(θ)=0, where

$$w_{i}^{k}={\left|a_{i}^{T}\theta^{k}-b_{i}\right|}^{p-2}.$$

Problem (10) is quadratic and $\theta^{k+1}$ can be computed in closed-form.

The direct alternating minimization algorithm is summarized as Algorithm 1.
**Algorithm 1:** AM Algorithm **Input:** A start point $\left(\theta_{0},s_{0}\right)$, and set β>0. **While** not converged (k=0,1,2,…) **do**     Update s by (6) for fixed $\theta_{k}$ to obtain $s_{k+1}$.     Update θ by (7) for fixed $s_{k+1}$ to obtain $\theta_{k+1}$. **End while** **Output:**$\left(\theta_{k+1},s_{k+1}\right)$.

#### 2.2.2. Alternating Minimization with Embedded Semidefinite Relaxation

The direct AM algorithm is efficient, but can easily get trapped in local minima due to the high nonconvexity of (1), which involves binary variables. In an attempt to alleviate this problem, we consider a relaxation of the binary variables by SDR. SDR has been shown to be very effective in handling combinatorial problems, and it is tighter than linear relaxation [40,41].

Let $\tilde{s}\in {\left\{-1,1\right\}}^{N+1}$, $S=\tilde{s}{\tilde{s}}^{T}$, $\Phi_{i}:=\Phi \left(r_{i}\left(\theta \right)\right)$, and

$$\Lambda =\left[\;\;\begin{array}{cc} 0 & (\beta -\Phi^{T})/2\\ (\beta -\Phi )/2 & \mathrm{diag}(\Phi ) \end{array}\;\;\right],$$

with $\Phi ={\left[\Phi_{1},\Phi_{2},\ldots ,\Phi_{N}\right]}^{T}$. Then, problem (1) is equivalent to

(11) $$\begin{array}{cc} \; & \;\;\;\;\;\;\;\;\;\;\;\;\;\;\underset{\theta \in R^{d},S\in S^{\left(N+1\right)}}{\min\;}\mathrm{tr}\left(\Lambda S\right)\\ \text{s.t.}\; & \mathrm{diag}\left(S\right)\;=\;1_{N+1},\;S⪰0,\;\mathrm{rank}\left(S\right)\;=\;1,\;h\left(\theta \right)\;=\;0. \end{array}$$

Then, dropping the rank-1 nonconvex constraint leads to a SDR of (1) as (derived in Appendix D)

(12) $$\begin{array}{cc} \; & \;\;\;\;\;\;\;\;\underset{\theta \in R^{d},S\in S^{\left(N+1\right)}}{\min\;}\mathrm{tr}\left(\Lambda S\right)\\ \text{s.t.} & \;\;\;\mathrm{diag}\left(S\right)=1_{N+1},\;\;S⪰0,\;\;h\left(\theta \right)\;=\;0. \end{array}$$

The above SDR is standard and widely used in binary combinatorial optimization problems [41], except that our problem additionally involves another variable θ needing to be solved simultaneously. As a consequence, unlike most existing well-studied SDR problems resulting in convex relaxed formulations, 12 is nonconvex. However, for some applications with Φ and $r_{i}$ being convex, problem (12) can be biconvex.

The problem (12) can be solved via alternatingly updating θ and S. Specifically, fixing S, problem (12) becomes

(13) $$\begin{array}{cc} \; & \underset{\theta \in R^{d}}{\min\;}\sum_{i=1}^{N}\;\left(1\;-\;S_{1,i+1}\right)\Phi \left(r_{i}\left(\theta \right)\right)\\ \; & \;\;\;\;\text{s.t.}\;\;\;\;h(\theta )\;=\;0, \end{array}$$

which is a form of (7). Fixing θ, the S-update subproblem becomes

(14) $$\begin{array}{cc} \; & \underset{S\in S^{\left(N+1\right)}}{\min\;}\mathrm{tr}\left(\Lambda S\right)\\ \text{s.t.}\; & \mathrm{diag}\left(S\right)=1_{N+1},\;\;S⪰0. \end{array}$$

It is a standard SDP and can be solved by well-established SDP solvers, such as CVX [42]. But such solvers using a prime-dual-interior algorithm have a complexity of $O({\left(N+1\right)}^{4.5})$ at the worst-case, and do not scale to moderate to large problem sizes.

In order to make the algorithm scalable to high-dimension problems, the low-rank property of S can be exploited by the Burer–Monteiro (B-M) factorization [41], e.g., using $S=RR^{T}$ with $R\in R^{(N+1)\times p}$ to recast (14) into

(15) $$\begin{array}{cc} \; & \underset{R\in R^{(N+1)\times p}}{\min\;}\mathrm{tr}\left(\Lambda RR^{T}\right)\\ \text{s.t.} & \;\mathrm{diag}\left(RR^{T}\right)=1_{N+1}. \end{array}$$

The B-M method is especially efficient to handle SDP problems, which has been applied in robotics and vision such as [43]. With this reformulation, the parameter number is reduced from ${\left(N+1\right)}^{2}$ to p(N+1). It has been proven that there exists an optimum of (14) with rank less than $\left\lceil \sqrt{2N}\right\rceil$ [41,44], hence using $p\;\geq \;\lceil \sqrt{2N}\rceil$ can guarantee that any optimum of (15) is also an optimum of (14). Meanwhile, although problem (15) is nonconvex, it almost never has any spurious local optima [45].

**Proposition** **3** ([45])**.** *For almost all ***Λ***, if p(p+1)≥2(N+1), any local optimum $R^{•}$ of (15) is a global optimum of (15), and $R^{•}R^{•T}$ is a global optimum of (14).*

This result implies that, despite the nonconvexity of (15), local optimization algorithms can converge to global optima. Accordingly, the first-order augmented Lagrangian algorithm [41] can be used. Rather than directly using this algorithm, we exploit the sparsity structure of the problem to achieve further acceleration. This is based on the fact that only the first column, first row and diagonal of Λ have nonzero elements. Specifically, let $r_{i}^{T}:=R\left(i,:\right)$ denote the *i*-th row of R and $r:={\left[r_{1}^{T},r_{2}^{T},\ldots ,r_{N+1}^{T}\right]}^{T}\in R^{p(N+1)}$, using the equivalence between $\mathrm{diag}\left(RR^{T}\right)=1_{N+1}$ and ${∥r_{i}∥}^{2}=1$ for 1≤i≤N+1, problem (15) can be reformulated as an unconstrained problem

(16) $$\underset{r\in R^{p(N+1)}}{\min\;}J\left(r\right):=2\sum_{i=2}^{N+1}\Lambda_{1,i}\frac{r_{1}^{T}r_{i}}{∥r_{1}∥∥r_{i}∥}$$

where the norm-one constraints are removed by changing variable to ${\left[R\left(i,:\right)\right]}^{T}=r_{i}/∥r_{i}∥$ [46], which leads to an unconstrained formulation.

Due to the equivalence between (15) and (16), and from Proposition 3, a local optimization algorithm can be employed to solve (16), and any solution that satisfies first- and second-order necessary optimality conditions is a global optimum. Hence, any efficient first-order algorithm can be employed, e.g., the limited memory BFGS (L-BFGS) algorithm [47]. Such algorithms only require evaluating the first-order gradient of the objective, which is

$$\nabla_{r}J\left(r\right)={\left[\nabla_{r_{1}^{T}}J\left(r\right),\nabla_{r_{2}^{T}}J\left(r\right),\ldots ,\nabla_{r_{N+1}^{T}}J\left(r\right)\right]}^{T},$$

with

$$\begin{array}{cc} \nabla_{r_{1}}J\left(r\right) & =2\sum_{i=2}^{N+1}\Lambda_{1,i}\frac{{∥r_{1}∥}^{2}r_{i}-r_{1}^{T}r_{i}r_{1}}{{∥r_{1}∥}^{3}∥r_{i}∥},\\ \nabla_{r_{i}}J\left(r\right) & =2\Lambda_{1,i}\frac{{∥r_{i}∥}^{2}r_{1}-r_{1}^{T}r_{i}r_{i}}{∥r_{1}∥{∥r_{i}∥}^{3}},\;\;\;\;\mathrm{for}\;2\leq i\leq N+1. \end{array}$$

The alternating minimization algorithm with SDR (AM-R) is summarized as Algorithm 2.
**Algorithm 2:** AM Algorithm with SDR (AM-R) **Input:** A start point $\left(\theta_{0},S_{0}\right)$, and set β>0. **While** not converged (k=0,1,2,…) **do**     Update S by (14) for fixed $\theta_{k}$ to obtain $S_{k+1}$.     Update θ by (13) for fixed $S_{k+1}$ to obtain $\theta_{k+1}$. **End while** **Output:**$\left(\theta_{k+1},S_{k+1}\right)$.

### 2.3. Application to 3D Registration

This section presents the application of SIME to rotation search and 6-DoF rigid registration. Rotation search, also known as the Wahba problem, aims to estimate the rotation between two coordinate frames, which has wide applications in computer vision, robotics, and aerospace engineering [48,49,50,51,52]. Six-DoF rigid registration can be viewed as an extension of 3-DoF rotation search, which estimates both rotation and translation.

#### 2.3.1. Rotation Search

Consider a set of 3D point pairs $\left\{\left(a_{i},b_{i}\right):i=1,2,\ldots ,N\right\}$ with $a_{i},b_{i}\in R^{3}$, which are generated as

(17) $$b_{i}=Ra_{i}+n_{i}+o_{i},$$

where R∈SO(3) is the unknown rotation, $n_{i}$ models small inlier measurement noise, and $o_{i}$ is zero if the data pair $(a_{i},b_{i})$ is an inlier, or $o_{i}$ is an arbitrary perturbation if $(a_{i},b_{i})$ is an outlier.

For convenience, we adopt quaternion representation for 3D rotation [53]. Denote a unit quaternion by $q={\left[v^{T}\;s\right]}^{T}$, where $v\in R^{3}$ is the vector part and *s* is the scalar part. If R is the unique rotation corresponding to a unit quaternion q, then the rotation of a vector $a\in R^{3}$ by R can be expressed in terms of the quaternion product as

$$\left[\;\;\begin{array}{c} \mathrm{Ra}\\ 0 \end{array}\;\;\right]=q\circ \hat{a}\circ q^{-1},$$

where $q^{-1}={\left[-v^{T}\;s\right]}^{T}$ is the quaternion inverse, and $\hat{a}={\left[a^{T}\;0\right]}^{T}$. The quaternion product is defined as q∘x=Ω(q)x for any $x\in R^{4}$, and $q_{1}\circ q_{2}=\Omega \left(q_{1}\right)q_{2}=\bar{\Omega }\left(q_{2}\right)q_{1}$ for two unit quaternions $q_{1}$ and $q_{2}$, where

$$\Omega \left(q\right)\;\;=\;\;\left[\;\;\;\begin{array}{cccc} q_{4}\; & \;-q_{3}\; & \;q_{2}\; & \;q_{1}\\ q_{3}\; & \;q_{4}\; & \;-q_{1}\; & \;q_{2}\\ -q_{2}\; & \;q_{1}\; & \;q_{4}\; & \;q_{3}\\ -q_{1}\; & \;-q_{2}\; & \;-q_{3}\; & \;q_{4} \end{array}\;\;\;\right]\;\;,\;\;\;\bar{\Omega }\left(q\right)\;\;=\;\;\left[\;\;\;\begin{array}{cccc} \;q_{4}\; & \;q_{3}\; & \;-q_{2}\; & \;q_{1}\\ \;-q_{3}\; & \;q_{4}\; & \;q_{1}\; & \;q_{2}\\ \;q_{2}\; & \;-q_{1}\; & \;q_{4}\; & \;q_{3}\\ \;-q_{1}\; & \;-q_{2}\; & \;-q_{3}\; & \;q_{4} \end{array}\;\;\;\right]\;\;.$$

Based on quaternion representation, the linear residual with a LS loss can be expressed as

(18) $$\Phi \left(r_{i}\left(\theta \right)\right)=∥{\hat{b}}_{i}-\theta \circ {\hat{a}}_{i}\circ \theta^{-1}{∥}^{2},\;\;\mathrm{with}\;\;∥\theta ∥=1.$$

For the AM-R algorithm, similar to (12), the SDR of SIME in this case leads to

(19) $$\begin{array}{cc} \; & \underset{\theta \in R^{4},S\in S^{\left(N+1\right)}}{\min\;}\mathrm{tr}\left(\Lambda S\right)\\ \text{s.t.} & \;\;\mathrm{diag}\left(S\right)=1_{N+1},\;S⪰0,\;∥\theta ∥\;=\;1. \end{array}$$

Accordingly, the θ-subproblem becomes

(20) $$\begin{array}{cc} \; & \underset{\theta \in R^{4}}{\min\;}\sum_{i=1}^{N}\left(1-S_{1,i+1}\right){∥{\hat{b}}_{i}-\theta \circ {\hat{a}}_{i}\circ \theta^{-1}∥}^{2}\\ \; & \;\;\;\;\text{s.t.}\;\;∥\theta ∥=1. \end{array}$$

For a unit quaternion θ, it follows that $c=\theta^{T}\left(cI_{4}\right)\theta$ for any c∈R, ${\hat{b}}_{i}^{T}\left(\theta \circ {\hat{a}}_{i}\circ \theta^{-1}\right)=\theta^{T}\Omega^{T}\left({\hat{b}}_{i}\right)\bar{\Omega }\left({\hat{a}}_{i}\right)\theta$ and $-\Omega^{T}\left({\hat{b}}_{i}\right)=\Omega \left({\hat{b}}_{i}\right)$, hence problem (20) can be rewritten as

(21) $$\underset{\theta \in R^{4}}{\min\;}\theta^{T}G\theta ,\;\;\;\;\text{s.t.}\;\;∥\theta ∥=1,$$

with

$$G=\sum_{i=1}^{N}\left(1-S_{1,i+1}\right)\left[\left({∥b_{i}∥}^{2}+{∥a_{i}∥}^{2}\right)I_{4}+2\Omega \left({\hat{b}}_{i}\right)\bar{\Omega }\left({\hat{a}}_{i}\right)\right].$$

Obviously, the solution of (21) is given by the eigenvector corresponding to the smallest eigenvalue of G.

Next, we compare the proposed method with a close existing method [30], namely QUASAR (QUAternion-based Semidefinite relAxation for Robust alignment). QUASAR uses a truncated LS loss and has a formulation as

(22) $$\underset{\begin{array}{c} \theta \in R^{4},∥\theta ∥=1\\ s_{i}\in \left\{-1,1\right\} \end{array}}{\min\;}\sum_{i=1}^{N}\frac{1-s_{i}}{2}\frac{{∥{\hat{b}}_{i}-\theta \circ {\hat{a}}_{i}\circ \theta^{-1}∥}^{2}}{\sigma^{2}}+\frac{1+s_{i}}{2}{\bar{c}}^{2},$$

which can be viewed as a special instance of the SIME formulation. To solve this mixed-integer program, QUASAR adopts a binary cloning based reformulation as

(23) $$\begin{array}{cc} \underset{\begin{array}{c} \theta \in R^{4},∥\theta ∥=1\\ \theta_{i}=\pm \theta \end{array}}{\min\;}\sum_{i=1}^{N} & \frac{{∥{\hat{b}}_{i}\;-\;\theta \;\circ \;{\hat{a}}_{i}\;\circ \;\theta^{-1}\;-\;\theta^{T}\theta_{i}{\hat{b}}_{i}\;+\;\theta \;\circ \;{\hat{a}}_{i}\;\circ \;\theta_{i}^{-1}∥}^{2}}{4\sigma^{2}}\;\\ \; & \;\;\;\;\;\;\;\;\;\;\;\;\;\;+\frac{1+\theta^{T}\theta_{i}}{2}{\bar{c}}^{2}. \end{array}$$

This reformulation is based on the fact that, if $\theta_{i}=s_{i}\theta$ with $s_{i}\in \left\{-1,1\right\}$, then $\theta^{T}\theta_{i}=s_{i}$ and $\theta \circ {\hat{a}}_{i}\circ \theta_{i}^{-1}=s_{i}\left(\theta \circ {\hat{a}}_{i}\circ \theta^{-1}\right)$. Let $\tilde{\theta }={\left[\theta^{T},\theta_{1}^{T},\ldots ,\theta_{N}^{T}\right]}^{T}$, then problem (23) can be expressed as [30]

(24) $$\begin{array}{cc} \; & \min_{\tilde{\theta }\in R^{4(N+1)}}\;{\tilde{\theta }}^{T}Q\tilde{\theta }\\ \text{s.t.}\;\; & ∥\theta ∥=1,\;\theta_{i}\theta_{i}^{T}=\theta \theta^{T},\;\forall i=1,\ldots ,N, \end{array}$$

where $Q\in R^{4(N+1)\times 4(N+1)}$ is given by

$$Q=\left[\;\;\begin{array}{cccc} 0 & Q_{01} & \ldots & Q_{0N}\\ Q_{01} & Q_{11} & \ldots & 0\\ \vdots & \vdots & \ddots & \vdots \\ Q_{0N} & 0 & \ldots & Q_{NN} \end{array}\;\;\right],$$

with

$$Q_{ii}=\frac{\left({∥b_{i}∥}^{2}+{∥a_{i}∥}^{2}\right)I_{4}+2\Omega \left({\hat{b}}_{i}\right)\bar{\Omega }\left({\hat{a}}_{i}\right)}{2\sigma^{2}}+\frac{{\bar{c}}^{2}}{2}I_{4},$$

$$Q_{0i}=-\frac{\left({∥b_{i}∥}^{2}+{∥a_{i}∥}^{2}\right)I_{4}+2\Omega \left({\hat{b}}_{i}\right)\bar{\Omega }\left({\hat{a}}_{i}\right)}{4\sigma^{2}}+\frac{{\bar{c}}^{2}}{4}I_{4}.$$

Then, let $Z=\tilde{\theta }{\tilde{\theta }}^{T}\in S^{4(N+1)}$ and denote its 4×4 sub-blocks by ${\left[Z\right]}_{ij}=\theta_{i}\theta_{j}^{T}$ for ∀0≤i,j≤N with $\theta_{0}=\theta$; QUASAR adopts a SDR of (24) as

(25) $$\begin{array}{cc} \; & \min_{Z\in S^{4(N+1)}}\mathrm{tr}\left(\mathrm{QZ}\right)\\ \text{s.t.}\;\; & \mathrm{tr}({\left[Z\right]}_{00})=1,\;Z⪰0,\\ \; & {\left[Z\right]}_{ii}={\left[Z\right]}_{00},\;\forall i=1,\ldots ,N,\\ \; & {\left[Z\right]}_{ij}={\left[Z\right]}_{ij}^{T},\;\forall 0\leq i<j\leq N.\;\;\;\; \end{array}$$

The next result compares QUASAR with the proposed AM-R algorithm.

**Proposition** **4.** *If the SIME formulation uses the loss (18) and with $\beta =\sigma^{2}{\bar{c}}^{2}$, then it is equivalent to the QUASAR formulations (22)–(24). Furthermore, the relaxation (19) of SIME is tighter than the relaxation (25) of QUASAR.*

**Proof.**  When the loss (18) and $\beta =\sigma^{2}{\bar{c}}^{2}$ are used in SIME, it is easy to see the equivalence between SIME and (22) by changing variable from $s_{i}\in \left\{0,1\right\}$ to $s_{i}\in \left\{-1,1\right\}$. To justify that (19) is tighter than (25), we first show that

(26) $$\mathrm{tr}\left(\Lambda S\right)=2\sigma^{2}\mathrm{tr}\left(Q\left(S⊗(\theta \theta^{T})\right)\right)-\sigma^{2}{\bar{c}}^{2}.$$

From the properties of the trace and Kronecker product operations, some algebra leads to $\mathrm{tr}\left(Q\left(S⊗(\theta \theta^{T})\right)\right)=\theta^{T}\bar{Q}\theta$ with

$$\bar{Q}=\sum_{i=1}^{N}Q_{ii}+2S_{1,i+1}Q_{0i}.$$

Similarly, with the loss (18) and $\beta =\sigma^{2}{\bar{c}}^{2}$, it follows that $\mathrm{tr}\left(\Lambda S\right)=2\sigma^{2}\theta^{T}\bar{Q}\theta -\sigma^{2}{\bar{c}}^{2}$, which leads to (26). Hence, the relaxation (19) is equivalent to

$$\begin{array}{cc} \; & \min_{\theta \in R^{4},S\in S^{\left(N+1\right)}}\;\mathrm{tr}\left(Q\left(S⊗(\theta \theta^{T})\right)\right)\\ \; & s.t.\;\;∥\theta ∥=1,\;\mathrm{diag}\left(S\right)=1_{N+1},\;S⪰0, \end{array}$$

which is further equivalent to

(27) $$\begin{array}{cc} \; & \min_{Z\in S^{4(N+1)}}\;\mathrm{tr}\left(\mathrm{QZ}\right)\\ \text{s.t.} & \;\;{\left[Z\right]}_{00}=\theta \theta^{T},\;∥\theta ∥=1,\\ \; & \;\;{\left[Z\right]}_{ii}={\left[Z\right]}_{00},\;\forall i=1,\ldots ,N,\\ \; & \;\;{\left[Z\right]}_{ij}=s_{ij}{\left[Z\right]}_{00},\;\forall 0\leq i<j\leq N,\\ \; & \;\;S=\left[\;\;\begin{array}{cccc} 1 & s_{01} & \ldots & s_{0N}\\ s_{01} & 1 & \ldots & s_{1N}\\ \vdots & \vdots & \ddots & \vdots \\ s_{0N} & s_{1N} & \ldots & 1 \end{array}\;\;\right]⪰0. \end{array}$$

Then, it is easy to see that the constraints in (27) in fact constitute a subset of the constraints in (25). Hence, the optimum objective of (27) (and equivalently that of (19)) provides a tighter lower bound to the original nonconvex problem than (25). □

**Remark** **4** (Computational complexity)**.**
*It has been shown in [30] that the relaxation (25) with redundant constraints is sufficiently tight. Particularly, in the noiseless and outlier-free case, it is always tight as its optimal solution has rank-1 and attains a global minimum of the original nonconvex problem. However, QUASAR solving (25) is computationally expensive and scales poorly in problem size. For example, with a general SDP solver, it typically needs more than 1000 s for N=100 [30]. Although (25) is also a SDP like the S-subproblem (14), the accelerating procedure in Section 3.2 does not apply to it. That is because the SDP (25) involves a large number of equality constraints, about $3N^{2}+13N$. Meanwhile, the constraints do not admit an unconstrained formulation. Hence, when using the augmented Lagrangian method, a large number of dual variables (about $3N^{2}+13N$) have to be handled, which fundamentally increases the computational complexity. In comparison, (14) only has N+1 equality constraints and, more importantly, the norm-one constraints have a special “hidden convexity” structure admitting an unconstrained formulation (16). This leads to a significant advantage of our algorithm over QUASAR in computational efficiency, e.g., three orders of magnitude faster, as will be shown in experiments.*

Moreover, for the AM algorithm, the θ-subproblem can be expressed as (20) with $\left(1\;-\;S_{1,i+1}\right)$ replaced by $\left(1\;\;-s_{i}\right)$, where $s_{i}$ is computed by (6).

When considering $\ell_{p}$ loss with 1≤p<2, the θ-subproblem (20) can be solved by the iteratively reweighted LS algorithm as

(28) $$\begin{array}{cc} \theta^{k+1} & =\arg\min_{\theta \in R^{4}}\sum_{i=1}^{N}\left(1-S_{1,i+1}\right)w_{i}^{k+1}{∥{\hat{b}}_{i}-\theta \circ {\hat{a}}_{i}\circ \theta^{-1}∥}^{2}\\ \; & \text{s.t.}\;\;∥\theta ∥=1, \end{array}$$

where

$$w_{i}^{k+1}={∥{\hat{b}}_{i}-\theta^{k}\circ {\hat{a}}_{i}\circ {\left(\theta^{k}\right)}^{-1}∥}^{p-2}.$$

Then, in each iteration, problem (28) can be solved in closed form via eigen-decomposition similar to (21).

#### 2.3.2. 6-DoF Euclidean Registration

In the context of 6-DoF Euclidean registration defined by a rigid transformation $\left[R,t\right]\in R^{3\times 4}$, where R∈SO(3) and t are the unknown rotation and translation, respectively, the generation model (17) is extended to

(29) $$b_{i}=Ra_{i}+t+n_{i}+o_{i}.$$

In this case, using quaternion representation for rotation and with the LS loss, the fitting objective becomes

(30) $$\Phi \left(r_{i}\left(\theta \right)\right)={∥{\hat{b}}_{i}-\theta \circ {\hat{a}}_{i}\circ \theta^{-1}-\hat{t}∥}^{2},\;\;\mathrm{with}\;\;∥\theta ∥=1,$$

where $\hat{t}={\left[t^{T}\;0\right]}^{T}$. Accordingly, the θ-subproblem becomes

(31) $$\begin{array}{cc} \; & \underset{\theta \in R^{4},t\in R^{3}}{\min\;}\sum_{i=1}^{N}\omega_{i}{∥{\hat{b}}_{i}\;-\;\theta \;\circ \;{\hat{a}}_{i}\;\circ \;\theta^{-1}\;-\;\hat{t}∥}^{2}\\ \; & \;\;\;\;\text{s.t.}\;\;∥\theta ∥=1. \end{array}$$

For the AM algorithm, $\omega_{i}=1-s_{i}$ with $s_{i}$ can be computed by (6), whilst for the AM-R algorithm, $\omega_{i}=1-S_{1,i+1}$ with $S_{i,j}$ can be computed by (14). Then, the closed-form solution to problem (31) is given in [54].

When using the $\ell_{p}$ loss with 1≤p<2 instead of the $\ell_{2}$ loss in (31), there is no closed-form solution. In this case, the θ-subproblem can be solved by the reweighted LS algorithm similar to (28).

## 3. Results

We evaluate the proposed algorithms in 3D registration experiments, including simulated 3-DoF rotation registration and 6-DoF Euclidean registration, and a real-world 3D registration experiment. For the AM-R algorithm (Algorithm 2), we set $p=\lceil \sqrt{2N}/3\rceil$ for the low-rank factorization (15) and solve (16) by L-BFGS using *minFunc* [55]. This choice of *p* is motivated by a practical trade-off between relaxation quality and computational efficiency. Although the sufficient rank condition for the Burer–Monteiro factorization requires $p\;\geq \;\lceil \sqrt{2N}\rceil$, we empirically found that a smaller rank is adequate for the sparse SDP subproblem considered here, while significantly reducing the number of optimization variables.

### 3.1. Rotation Registration

In the first experiment, we evaluate the new algorithms on rotation search, in comparison with (i) RANSAC; (ii) GORE-RANSAC, which uses GORE [56] to firstly remove most outliers and then uses RANSAC to estimate the model from the pruned measurements; (iii) Fast global registration (FGR) [29]; and (iv) QUASAR [30], which is implemented in Matlab using CVX [42] with MOSEK [57] as the SDP solver. Note that GORE can provide a rough estimation of the model with guaranteed outlier removal, but GORE-RANSAC has significantly better accuracy in most cases. For QUASAR, AM and AM-R, we use the same noise bound parameter $\beta ={\bar{c}}^{2}\sigma^{2}$ such that $P\left({∥b_{i}-Ra_{i}∥}^{2}\leq {\bar{c}}^{2}\sigma^{2}\right)=1-10^{-6}$ holds for inliers, which under Gaussian inlier noise can be computed from the 3-DoF Chi-squared distribution. For the rotation registration problem, AM alternatingly solves the two subproblems (6) and (20), whilst AM-R alternatingly solves the two subproblems (14) and (20). We use the RANSAC solution as the initialization of AM and AM-R. All the runtime results of AM and AM-R include the runtime of the RANSAC initialization.

The Bunny dataset from the Stanford 3D Scanning Repository [58] is used. Firstly, the point cloud is resized into a unit cube ${\left[0,1\right]}^{3}$ and randomly down-sampled to *N* points with N∈{100,500}. Then, a random rotation is applied and additive noise and outliers are randomly generated according to (17). Two conditions with low and high inlier noise are considered, with σ=0.01 and σ=0.1, respectively. Meanwhile, different outlier ratios from 0 to 95% are considered. Each result is an average of 50 independent runs.

**Figure 1:**
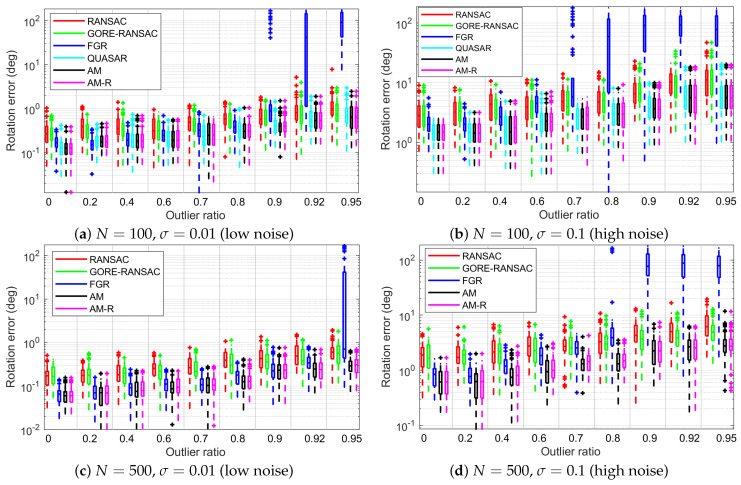
Rotation error comparison in the rotation registration experiment with low and high noise conditions. QUASAR is not compared in the case of N=500 as it runs out of memory when N>150.

Figure 1 presents the rotation error of the algorithms in the two noise conditions for N=100 and N=500, respectively. It can be seen that FGR performs well at low outlier ratios, but tends to beak at relatively high outlier ratios, e.g., when the outlier ratio exceeds 70% in the case of N=100 and σ=0.1. GORE-RANSAC generally has better performance than RANSAC as it firstly removes most of the outliers by the GORE method. For N=100, QUASAR, AM and AM-R generally perform comparably and outperform the others. In the case of N=500, QUASAR is not compared as it runs out of memory when N>150. For N=500, SIME distinctly outperforms its counterparts in most cases, and the advantage is especially conspicuous in the high noise case.

**Figure 2:**
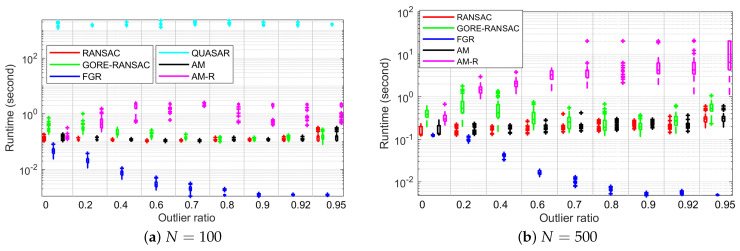
Runtime comparison in the rotation registration experiment with σ=0.1.

Figure 2 compares the runtime of the algorithms. Clearly, FGR is the fastest. Though both QUASAR and AM-R involve solving SDP, AM-R is about 1000 times faster than QUASAR in the case of N=100. This is thanks to the accelerating strategy using B-M factorization and the unconstrained formulation exploiting the sparsity of the problem. However, QUASAR cannot be accelerated like AM-R as it has a large number of constraints as explained in Remark 4.

### 3.2. 6-DoF Euclidean Registration

In the second experiment, we consider 6-DoF rigid registration, where both rotation and translation need to be estimated. We generate the data measurements similar to the rotation registration experiment except that a random translation is additionally considered according to (29). In this setting, AM alternatingly solves the two subproblems (6) and (31), whilst AM-R alternatingly solves the two subproblems (14) and (31). The TEASER++ algorithm (implemented in C++) [31] is also compared in this experiment. TEASER++ decouples the scale, rotation and translation estimation and solves them separately and sequentially. It solves decoupled scale and translation estimation via adaptive voting, and solves the rotation estimation via a graduated nonconvexity scheme [28], which has shown highly effectiveness and efficiency. The noise bound parameter of TEASER++ is tuned to provide the best performance.

**Figure 3:**
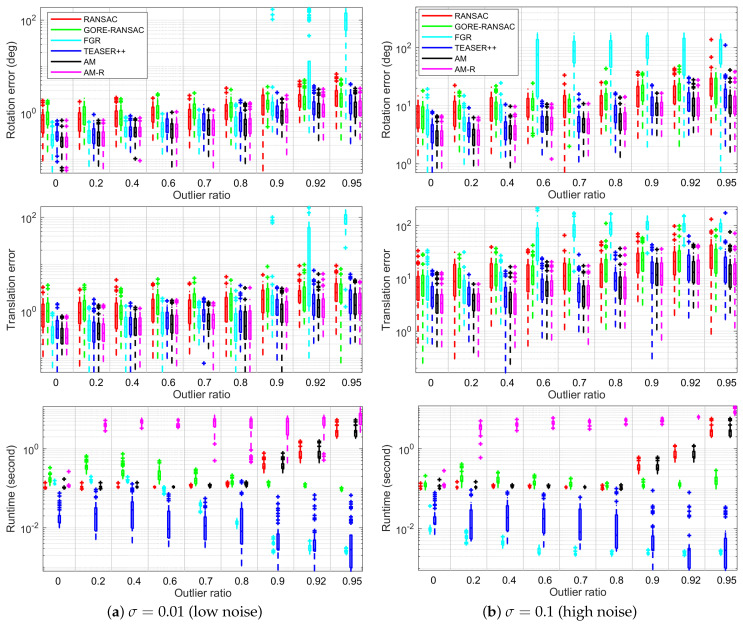
Rotation error, translation error and runtime comparison in the 6-DoF Euclidean registration experiment.

**Figure 4:**
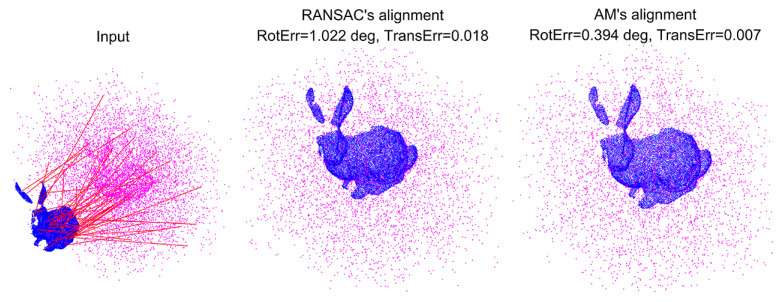
Illustration of 6-DoF Euclidean registration by RANSAC and the proposed AM algorithm (with outlier ratio being 80%), along with the rotation error (RotErr) and translation error (TransErr).

Figure 3 presents the rotation error, translation error and runtime of the compared algorithms for N=200. Similar to the rotation registration experiment, two noise conditions with σ=0.01 and σ=0.1 are considered. It can be seen that FGR tends to break at high outlier ratios, especially in the high noise condition, e.g., when the outlier ratio exceeds 50%. AM and AM-R achieve the best accuracy in most cases, and the advantage gets more prominent in the high noise condition. They again significantly outperform the RANSAC and GORE-RANSAC methods. Moreover, the results demonstrate the high efficiency of FGR and TEASER++, which are much faster than AM-R. Figure 4 illustrates a typical registration example by RANSAC and AM at an outlier ratio of 80%.

### 3.3. Truncated $\ell_{p}$ Loss

The above experiment considers the truncated LS loss. This experiment further investigates the truncated $\ell_{p}$ loss with 1≤p≤2. The inlier noise is generated as generalized Gaussian distribution (GGD) with zero-mean as

(32) $$p\left(x;v,\alpha \right)=\frac{v}{2\alpha \Gamma (1/v)}\exp\left(-\frac{{\left|x\right|}^{v}}{\alpha^{v}}\right),$$

where *v* is the shape parameter, α is the scale parameter, and Γ is the gamma function. GGD adapts to a large family of symmetric distributions, spanning from Laplace (v=1) to Gaussian (v=2). When v<2, the noise is super-Gaussian.

**Figure 5:**
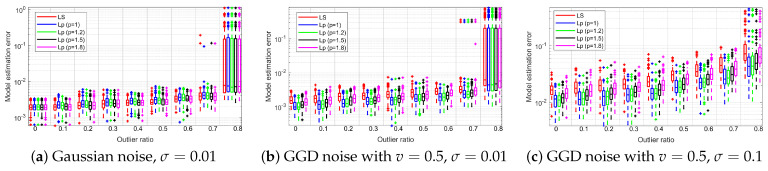
Performance of the AM algorithm with truncated LS or $\ell_{p}$ loss in the linear regression experiment in the case of GGD inlier noise (with shape parameter *v* and standard deviation σ) and uniformly distributed outliers in [−2, 2].

**Figure 6:**
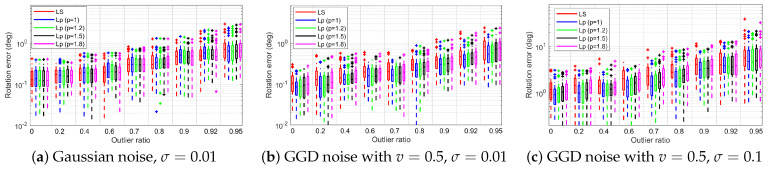
Performance of the AM algorithm with truncated LS or $\ell_{p}$ loss in the rotation registration experiment (with N=100) in the case of GGD inlier noise (with shape parameter *v* and standard deviation σ).

We consider two noise strengths with σ∈{0.01,0.1}. Figure 5 shows the performance of the AM algorithm using the truncated LS and truncated $\ell_{p}$ loss in the linear regression experiment, whilst Figure 6 shows that of the rotation registration experiment with N=100. Four different values of *p* are evaluated for the $\ell_{p}$ loss, with p∈{1,1.2,1.5,1.8}. Two cases with Gaussian and GGD (v=0.5) inlier noise are considered.

It can be seen from Figure 5 and Figure 6 that in the case of GGD noise with v=0.5, the truncated $\ell_{p}$ loss with a relatively small value of *p* (e.g., p=1) can yield significantly better accuracy than the truncated LS loss.

### 3.4. Real-World 3D Registration Experiment

We further evaluate the proposed SIME (AM) on a real-world scan matching task using the 3DMatch benchmark [59], which contains real indoor RGB-D scans from eight test scenes. For each scan, there are 5000 keypoints, on which putative correspondences are generated by nearest-neighbor matching on 3DSmoothNet descriptors [60]. We compare the proposed SIME (AM) with RANSAC and TEASER++ on the same correspondence sets. A registration result is counted as successful if the estimated rigid transformation has a rotation error smaller than $10^{\circ }$ and a translation error smaller than 30 cm. For RANSAC, we set the maximum number of iterations to 10,000. For SIME (AM), we use the RANSAC estimate as initialization.

Table 1 reports the percentage of correctly registered scan pairs for each test scene, together with the average runtime per scan pair. The runtime only includes the robust transformation estimation stage, since feature extraction and descriptor matching are identical for all compared methods. As can be seen, SIME (AM) achieves the best overall performance on the 3DMatch scan matching benchmark. Compared with TEASER++, SIME (AM) improves the mean registration success rate by 0.3%, while compared with RANSAC the gain reaches 4.3%. This result is so consistent with our theoretical analysis and synthetic experiments that by jointly selecting inliers and refining the model under a truncated-loss objective, SIME is able to obtain a more reliable consensus set and a lower fitting residual than methods based purely on consensus maximization.

The advantage of SIME is more evident in the controlled high-noise and high-outlier experiments, where residual-aware inlier selection can achieve more accurate model fitting. In the 3DMatch experiment, the gain over TEASER++ is smaller and comes with additional runtime due to the AM refinement step. This real-world result should be interpreted as demonstrating competitive performance over existing strong solvers. It is partly because the 3DMatch setting is affected by descriptor quality and correspondence generation, and TEASER++ already performs strongly on this benchmark.

## 4. Discussion

The results show that the proposed truncated-loss based SIME framework is an effective alternative to conventional MC-based robust registration methods. Unlike MC, which only counts whether a correspondence satisfies a residual threshold, SIME also considers the residual magnitude during inlier selection. This difference is important in 3D registration, where inlier identification and transformation estimation are strongly coupled. The experimental results are consistent with the theoretical analysis, showing that SIME can achieve more accurate registration, especially under high noise and high outlier ratios.

This work still has some limitations. The AM algorithm depends on a good initialization, and the SDR-based solver is more computationally expensive than lightweight registration methods. In addition, the current framework is mainly designed for rigid registration with tentative correspondences, and does not explicitly address non-rigid deformation. Future work will focus on improving scalability and extending the framework to more general registration problems.

This work has several limitations. First, SIME depends on the truncation parameter β, or equivalently the threshold τ when β=Φ(τ). A too large threshold may include true outliers, while a too small one may reject true inliers. In practice, τ can be selected according to the expected inlier noise level, but adaptive threshold selection remains future work. Second, although AM-R improves robustness by relaxing the binary inlier variables with SDR, it is more computationally expensive than the direct AM solver. Finally, as a nonconvex AM-based method, the proposed algorithm may be affected by initialization in extremely noisy or ambiguous cases.

## 5. Conclusions

This paper investigated a truncated-loss based formulation for robust 3D registration using simultaneous inlier identification and model estimation (SIME). Compared with maximum consensus methods that only rely on hard inlier counting, SIME enables lower achievable fitting residuals in robust model fitting. To solve the resulting nonconvex problem, we developed two alternating minimization methods, namely a direct AM algorithm and an SDR-enhanced AM-R algorithm. We instantiated the proposed framework for both rotation registration and 6-DoF Euclidean registration. Experimental results on synthetic and real-world data demonstrated that the proposed methods achieve strong robustness under high noise and high outlier ratios, and compare favorably with representative robust registration methods. Quantitatively, in the synthetic experiments, the proposed methods were evaluated under outlier ratios up to 95% and consistently achieved competitive or better accuracy, with particularly clear advantages in high-noise settings. For rotation registration with N=100, AM-R was about three orders of magnitude faster than QUASAR while maintaining comparable accuracy. On the real-world 3DMatch benchmark, SIME (AM) achieved the best mean registration success rate of 91.0%.

## Figures and Tables

**Table 1:** Percentage of correct registration results on the 3DMatch scan matching benchmark (%).

Method	Kitchen	Home 1	Home 2	Hotel 1	Hotel 2	Hotel 3	Study	MIT Lab	Mean	Avg. Runtime (ms)
RANSAC	96.2	91.7	74.5	91.6	84.6	90.7	82.2	81.8	86.7	101.3
TEASER++	97.8	92.3	82.7	96.9	88.5	94.4	88.7	84.4	90.7	87.4
SIME (AM)	98.2	92.3	83.2	97.3	88.5	94.4	89.4	84.4	91.0	112.9

## Data Availability

The data used in this work are publicly available at http://www-graphics.stanford.edu and https://3dmatch.cs.princeton.edu/ (accessed on 2 June 2022).

## Appendix A. Proof of Proposition 1

**Proof.** Let P∈(0,1] and 1−P∈[0,1) denote the prior probabilities of inliers and outliers, respectively. Then, under conditions (i) and (ii), the mixture density of $r_{i}$ is

$$p\left(r_{i}|\theta \right)=Pc_{i}e^{-\Phi (r_{i},\sigma_{i})}+\left(1-P\right)\frac{1}{u-\tau }I\left(\tau <r_{i}\leq u\right).$$

Thus, under conditions (i) and (ii), the likelihood of $r={\left[r_{1},r_{2},\ldots ,r_{N}\right]}^{T}$ is given by

$$\begin{array}{c} L\left(r;\theta \right)=\prod_{i=1}^{N}{\left[P\exp\left(-\Phi (r_{i},\sigma_{i})\right)\right]}^{1-I(\tau <r_{i}\leq u)}\\ \;\;\;\;\;\;\;\;\;\;\;\;\;\;\;\;\;\;\;\;\times {\left[P\exp\left(-\Phi (r_{i},\sigma_{i})\right)+\frac{1-P}{u-\tau }\right]}^{I(\tau <r_{i}\leq u)}, \end{array}$$

with h(θ)=0. The corresponding log-likelihood is

(A1) $$\begin{array}{cc} \; & \log L\left(r;\theta \right)=\sum_{i=1}^{N}\left(1-I(\tau <r_{i}\leq u)\right)\left[-\Phi (r_{i},\sigma_{i})+\log P\right]\\ \; & \;\;\;\;+I(\tau <r_{i}\leq u)\underset{L_{o}\left(r_{i}\right)}{\underset{︸}{\log\left[P\exp\left(-\Phi (r_{i},\sigma_{i})\right)+\frac{1-P}{u-\tau }\right]}}, \end{array}$$

with h(θ)=0. Since Φ is an increasing function, for $r_{i}\in \left(\tau ,u\right]$ it follows that

$$\log\left(1-P\right)-\log\left(u-\tau \right)<L_{o}\left(r_{i}\right)<L_{o}\left(\tau \right).$$

That is, as $r_{i}$ increases on (τ,u], $L_{o}\left(r_{i}\right)$ decreases from $L_{o}\left(\tau \right)$ toward log(1−P)−log(u−τ). Meanwhile, for sufficiently large *u*, we have $\exp\left(-\Phi (r_{i},\sigma_{i})\right)\to 0$ for $r_{i}>u$. Hence, the likelihood is dominated by the two cases of $r_{i}\leq \tau$ and $\tau <r_{i}\leq u$, i.e., $r_{i}\leq u$. Then, from the fact that $I(\tau <r_{i}\leq u)$ is equivalent to $I(\Phi \left(\tau \right)<\Phi \left(r_{i}\right)\leq \Phi \left(u\right))$, it is easy to see that with some β satisfying $\log\left(1-P\right)-\log\left(u-\tau \right)\leq \beta \leq L_{o}\left(\tau \right)$, the minimization problem,

$$\underset{\theta \in R^{d},s_{i}\in \left\{0,1\right\}}{\min\;}\sum_{i=1}^{N}\left(1-s_{i}\right)\Phi \left(r_{i},\sigma_{i}\right)+\beta s_{i},$$

can be viewed as an approximation of maximizing the log-likelihood (A1). Under i.i.d assumption of inliers with $\sigma_{i}=\sigma$ for $\forall i\in I^{*}$, taking the scale parameter σ into β, and ignoring constant terms independent on $r_{i}$, it finally leads to (1). □

## Appendix B. Proof of Proposition 2

**Proof.** Let $\left(\theta^{+},s^{+}\right)$ be a global solution of SIME, and let

$$I^{+}:=\left\{i:s_{i}^{+}=0\right\}$$

be its inlier set. Since, for fixed $\theta^{+}$, each binary variable $s_{i}^{+}$ minimizes

$$\left(1-s_{i}\right)\Phi \left(r_{i}\left(\theta^{+}\right)\right)+\Phi \left(\tau \right)s_{i}\;\mathrm{over}\;s_{i}\in \left\{0,1\right\},$$

it follows that

$$\Phi \left(r_{i}\left(\theta^{+}\right)\right)\leq \Phi \left(\tau \right),\;\forall i\in I^{+}.$$

Because Φ is increasing on [0,+∞), we have

$$r_{i}\left(\theta^{+}\right)\leq \tau ,\;\forall i\in I^{+}.$$

Hence $I^{+}$ is a consensus set. Moreover, since $I^{*}$ is a maximum consensus set, it follows that

$$|I^{+}\left|\leq \right|I^{*}|.$$

Now assume that $\Phi \left(r_{i}\left(\theta_{I^{*}}\right)\right)\leq \Phi \left(\tau \right)$ for all $i\in I^{*}$. Then, by monotonicity of Φ,

$$r_{i}\left(\theta_{I^{*}}\right)\leq \tau ,\;\forall i\in I^{*}.$$

Moreover, since $I^{*}$ is a maximum consensus set, there cannot exist any $j\in \Omega \setminus I^{*}$ such that

$$r_{j}\left(\theta_{I^{*}}\right)\leq \tau ,$$

otherwise $I^{*}\cup \left\{j\right\}$ would be a larger consensus set, contradicting the maximality of $I^{*}$. Therefore,

$$r_{j}\left(\theta_{I^{*}}\right)>\tau ,\;\forall j\in \Omega \setminus I^{*},$$

and hence

$$\Phi \left(r_{j}\left(\theta_{I^{*}}\right)\right)>\Phi \left(\tau \right),\;\forall j\in \Omega \setminus I^{*}.$$

Thus we have $I^{*}\in Ϝ$, and

$$f\left(\theta_{I^{*}},s_{I^{*}}\right)=R\left(I^{*}\right)+|\Omega \setminus I^{*}|\Phi \left(\tau \right).$$

For any other consensus set $I^{•}\in Ϝ$, we similarly have

$$f\left(\theta_{I^{•}},s_{I^{•}}\right)=R\left(I^{•}\right)+|\Omega \setminus I^{•}|\Phi \left(\tau \right).$$

Condition (4) is equivalent to

$$R\left(I^{•}\right)+|\Omega \setminus I^{•}|\Phi \left(\tau \right)>R\left(I^{*}\right)+|\Omega \setminus I^{*}|\Phi \left(\tau \right),$$

that is,

$$f\left(\theta_{I^{•}},s_{I^{•}}\right)>f\left(\theta_{I^{*}},s_{I^{*}}\right),\;\forall I^{•}\in Ϝ,\;I^{•}\neq I^{*}.$$

Therefore, $\left(\theta_{I^{*}},s_{I^{*}}\right)$ attains the minimum objective value of SIME, and the corresponding inlier set is $I^{*}$. Hence $I^{+}=I^{*}$. □

## Appendix C. Proof of Theorem 1

**Proof.** Let $\left(\theta^{+},s^{+}\right)$ be a global solution of SIME, and let

$$I^{+}:=\left\{i:s_{i}^{+}=0\right\}$$

be its inlier set. By Proposition 2, $I^{+}$ is a consensus set, and hence

$$|I^{+}\left|\leq \right|I^{*}|.$$

Moreover, since $\left(\theta^{+},s^{+}\right)$ is globally optimal for SIME, $\theta^{+}$ must minimize

$$\sum_{i\in I^{+}}\Phi \left(r_{i}\left(\theta \right)\right)$$

subject to h(θ)=0 for the fixed support $I^{+}$. Therefore,

$$\theta^{+}=\theta_{I^{+}},$$

and

$$f\left(\theta^{+},s^{+}\right)=R\left(I^{+}\right)+|\Omega \setminus I^{+}|\Phi \left(\tau \right).$$

On the other hand, by definition of $\theta_{I^{*}}$ and $s_{I^{*}}$, we have

$$f\left(\theta_{I^{*}},s_{I^{*}}\right)=R\left(I^{*}\right)+|\Omega \setminus I^{*}|\Phi \left(\tau \right).$$

Since $\left(\theta^{+},s^{+}\right)$ is a global solution of SIME, we have

$$f\left(\theta^{+},s^{+}\right)\leq f\left(\theta_{I^{*}},s_{I^{*}}\right),$$

that is,

$$R\left(I^{+}\right)+|\Omega \setminus I^{+}|\Phi \left(\tau \right)\leq R\left(I^{*}\right)+|\Omega \setminus I^{*}|\Phi \left(\tau \right).$$

Using $|I^{+}\left|\leq \right|I^{*}|$, equivalently $|\Omega \setminus I^{+}|\geq |\Omega \setminus I^{*}|$, it follows that

$$R\left(I^{+}\right)\leq R\left(I^{*}\right)-\left(|I^{*}\left|-\right|I^{+}|\right)\Phi \left(\tau \right)\leq R\left(I^{*}\right).$$

By the definitions of $R(I^{+})$ and $R(I^{*})$, this proves (5). Next, suppose that $I^{*}\notin Ϝ$. Then, by definition of Ϝ, the binary vector $s_{I^{*}}$ is not an optimal choice for the fixed parameter $\theta_{I^{*}}$. Hence there exists some $\tilde{s}\in {\left\{0,1\right\}}^{N}$ such that

$$f\left(\theta_{I^{*}},\tilde{s}\right)<f\left(\theta_{I^{*}},s_{I^{*}}\right).$$

Since $\left(\theta^{+},s^{+}\right)$ is globally optimal for SIME, we further have

$$f\left(\theta^{+},s^{+}\right)\leq f\left(\theta_{I^{*}},\tilde{s}\right)<f\left(\theta_{I^{*}},s_{I^{*}}\right).$$

Therefore,

$$R\left(I^{+}\right)+|\Omega \setminus I^{+}|\Phi \left(\tau \right)<R\left(I^{*}\right)+|\Omega \setminus I^{*}|\Phi \left(\tau \right).$$

With the use of $|\Omega \setminus I^{+}|\geq |\Omega \setminus I^{*}|$, we have

$$R\left(I^{+}\right)<R\left(I^{*}\right)-\left(|I^{*}\left|-\right|I^{+}|\right)\Phi \left(\tau \right)\leq R\left(I^{*}\right).$$

Hence the inequality in (5) is strict. □

## Appendix D. Derivation of (12)

To show the equivalence between the SIME formulation (1) and the formulation (12), it is enough to show the equivalence between

(A2) $$\min_{\theta \in R^{d},\;s\in {\left\{0,1\right\}}^{N}}\sum_{i=1}^{N}\left(1-s_{i}\right)\Phi \left(r_{i}\left(\theta \right)\right)+\beta s_{i},$$

and

(A3) $$\begin{array}{cc} \; & \underset{\theta \in R^{d},S\in S^{\left(N+1\right)}}{\min\;}\mathrm{tr}\left(\Lambda S\right)\\ \mathrm{subject}\;\mathrm{to}\;\; & \mathrm{diag}\left(S\right)=1_{N+1},S⪰0,\mathrm{rank}\left(S\right)=1, \end{array}$$

where

$$\Lambda =\left[\begin{array}{cc} 0 & (\beta -\Phi^{T})/2\\ (\beta -\Phi )/2 & \mathrm{diag}(\Phi ) \end{array}\right],$$

with

$$\Phi ={\left[\Phi_{1},\Phi_{2},\ldots ,\Phi_{N}\right]}^{T},\;\Phi_{i}:=\Phi \left(r_{i}\left(\theta \right)\right).$$

First, problem (A2) can be equivalently expressed as

(A4) $$\min_{\theta \in R^{d},s\in {\left\{-1,1\right\}}^{N}}\frac{1}{2}\sum_{i=1}^{N}\left(1-s_{i}\right)\Phi \left(r_{i}\left(\theta \right)\right)+\left(1+s_{i}\right)\beta ,$$

where we changed $s\in {\left\{0,1\right\}}^{N}$ to $s\in {\left\{-1,1\right\}}^{N}$. Then, ignoring the constant terms, it is easy to see that problem (A4) can be equivalently expressed as

(A5) $$\min_{\theta \in R^{d},s\in {\left\{-1,1\right\}}^{N}}{\left(\beta -\Phi \right)}^{T}s+s^{T}\mathrm{diag}\left(\Phi \right)s.$$

Let

$$\bar{s}={\left[t,s^{T}\right]}^{T}\in {\left\{-1,1\right\}}^{N+1},$$

then the objective of (A5) can be rewritten as ${\bar{s}}^{T}\Lambda \bar{s}$. Here, an auxiliary variable t∈{−1,1} is introduced, with which s/t will be the solution. Meanwhile, define

$$S:=\bar{s}{\bar{s}}^{T}.$$

It is easy to see that $S=\bar{s}{\bar{s}}^{T}$ with constraint $\bar{s}\in {\left\{-1,1\right\}}^{N+1}$ is equivalent to

$$\mathrm{diag}\left(S\right)=1_{N+1},\;S⪰0,\;\mathrm{rank}\left(S\right)=1.$$

Thus, with ${\bar{s}}^{T}\Lambda \bar{s}=\mathrm{tr}\left(\Lambda S\right)$, problem (A5) can be rewritten as (A3), which justifies the equivalence between (A2) and (A3), and finally leads to (12).
